# Correlation between the liver transection line localization and future liver remnant hypertrophy in associating liver partition and portal vein ligation for staged hepatectomy

**DOI:** 10.3389/fsurg.2024.1369962

**Published:** 2024-05-27

**Authors:** Ivan Romic, Goran Augustin, Goran Pavlek, Elvira Kresic

**Affiliations:** ^1^ Department of Surgery, University Hospital Centre Zagreb, Zagreb, Croatia; ^2^ School of Medicine, University of Zagreb, Zagreb, Croatia; ^3^ Department of Radiology, University Hospital Centre Zagreb, Zagreb, Croatia

**Keywords:** ALPPS, hypertrophy, surgery, transection, remnant

## Abstract

**Background and aims:**

Colorectal liver metastases (CRLMs) represent the most prevalent form of secondary liver tumors, and insufficient future liver remnant (FLR) often leads to unresectability. To tackle this challenge, various methods for stimulating liver hypertrophy have been developed including portal vein embolization (PVE), associating liver partition and portal vein ligation for staged hepatectomy (ALPPS) and the newest one, liver venous deprivation (LVD). ALPPS was thoroughly studied over the last decade and it has been shown to induce rapid and intensive FLR hypertrophy. The objective of this study was to assess whether the localization of the liver transection line during the initial stage of ALPPS correlates with the degree of FLR hypertrophy.

**Methods:**

A retrospective, multicentric study was conducted, and we analyzed all consecutive patients with CRLMs who underwent ALPPS over the eight-year period. Patients were categorized into two groups based on the type of resection—right trisectionectomy (ERH) or right hemihepatectomy (RH) respectively. The degree of hypertrophy (DH), its correlation with FLR and postoperative outcomes were assessed.

**Results:**

The cohort consisted of 136 patients (72 in the ERH group and 64 in the RH group). Baseline characteristics, hypertrophy interval, and total liver volume showed no significant differences between the groups. DH was greater in the ERH group (83.2% vs. 62.5%, *p* = 0.025). A strong negative correlation was observed between FLR volume and DH in both groups. Postoperative outcomes and one-year survival were comparable between the groups.

**Conclusions:**

FLR hypertrophy is influenced by the localization of the liver transection line in ALPPS. Furthermore, correlation analysis indicated that a smaller estimated FLR is associated with greater DH. No statistical difference in outcomes was noted between the groups.

## Introduction

Colorectal liver metastases (CRLMs) are the most common secondary liver tumors. The presence of CRLMs represents a poor prognostic factor in patients with colorectal cancer (1). However, cure and long-term survival are achievable today, and beside surgical resection as the mainstay of CRLM treatment, there are other options such as liver transplantation, locoregional methods, or stereotactic body radiation therapy, which may be curative in selected patients. Standard liver resections are frequently contraindicated due to the risk of post-hepatectomy liver failure (PHLF), primarily stemming from inadequate functional liver parenchyma remnant. Hence, several methods have been developed to stimulate future liver remnant (FLR) hypertrophy, and one of these is “Associating liver partition and portal vein ligation for staged hepatectomy” (ALPPS), which has received a lot of attention in the surgical community over the last decade. Previous research has identified tumor-related factors, liver parenchymal condition, and intraoperative characteristics influencing FLR hypertrophy in ALPPS (2–4). However, additional research is necessary to clarify the exact mechanism underlying such intense and rapid FLR hypertrophy.

ALPPS combines portal vein ligation with two-stage hepatectomy (TSH). The initial stage involves liver division along the desired plane and ligation of the correspondent portal vein branch. The partitioned liver remains in the abdomen for 1–2 weeks, resulting in significant FLR hypertrophy. Follow-up volumetry typically occurs at the end of the first postoperative week, and if adequate FLR hypertrophy is observed, the second stage of ALPPS commences. In the second stage, the deportalized liver lobe is removed following the division of the corresponding portal pedicle.

German surgeon Hans Schlitt conducted the first ALPPS procedure in 2007 (5). Since then, ALPPS has evolved into the standard method for initially unresectable CRLMs in most European hepatobiliary centers (6, 7). In the beginning, concerns about safety were prominent since the mortality rate was reported to be 12%–15%, with morbidity rate exceeding 30% (7, 8). However, recent advancements in patient selection and technical improvements have significantly reduced postoperative complication rate to levels comparable to standard major liver resections (6, 9). The ALPPS registry was established in 2012 at the Zurich Clinical Hospital Center (Switzerland) with the aim of producing high-quality retrospective and prospective research on the safety and efficacy of ALPPS through multicentric/multinational cooperation. A secure online platform enables licensed surgeons to input patient data and exchange radiological images, volumetric data, and other clinical information. Patient data is anonymized, and personal information remains inaccessible. The research received approval from the scientific committee of the ALPPS registry on April 2, 2015. At the Department of Surgery, Division of Hepatobiliary Surgery and Transplantation of Abdominal Organs at the University Hospital Center Zagreb, the first ALPPS procedure was performed in 2015. Subsequently, it was critically evaluated and utilized for selected patients with bilobar liver malignancies and inadequate FLR.

The variability in segmental liver volumes complicates the planning of major liver resections due to the difficulties in predicting adequate FLR. Therefore, preoperative liver evaluation, including volumetry, is recommended for both standard hemihepatectomies and ALPPS. With CT volumetry, it is essential to be familiar with hepatic segmentation, and surgeon should be trained to identify liver segments and planned liver transection lines on axial imaging slices.

The primary objective of this multi-institutional study was to examine the correlation between the transection line and FLR hypertrophy in ALPPS using CT or MR volumetry in patients with CRLMs in a non-cirrhotic liver. Secondary objectives included the analysis of total and regional liver volumes and volume changes between the two stages of ALPPS, describing the operative characteristics of ALPPS, and defining the correlation between preoperative volumetric parameters and the degree of FLR hypertrophy. Additionally, early and one-year postoperative outcomes were analyzed.

## Methods and materials

### Research design and variable definition

A multicenter, retrospective cohort study of consecutive patients treated at high-volume hepatobiliary centers was conducted. Patient data from the University Hospital Centre Zagreb was extracted from the hospital information system, and the ethics committee approved the use of this data for research purposes (approval number: 19177-2/02/21-JG).

Personal patient data has been anonymized. Demographic and anthropometric data, liver, primary tumor, and CRLM characteristics, total and regional liver volumes were collected. The surgical technique followed the standardized ALPPS procedure (as described in the introduction). No additional radiological or laboratory tests were conducted for the purpose of this study.

The study included patients with CRLM who had a minimum of 12 months of follow-up. Inclusion criteria were: age ≥18 years; radiologically confirmed multiple CRLMs (with FLR/TLV < 30% or FLR/BW ratio <0.5); pathologically confirmed primary colorectal carcinoma (CRC); completion of both stages of ALPPS; and at least two (CT or MRI) abdominal scans with IV contrast, one for preoperative staging and the other before the second stage.

Exclusion criteria were: age <18 years; metastases of non-colorectal or non-carcinoma colorectal tumors (e.g., NET or GIST); incomplete ALPPS; ALPPS variations (laparoscopic, radiofrequency, or “Tourniquet ALPPS”); previous major liver resections or portal vein embolization (PVE); and presence of cirrhosis or portal hypertension.

Patients were categorized into two groups based on the localization of the transection line: the first line divided left lateral and left medial section along the falciform ligament, which corresponds to right trisectionectomy (extended right hepatectomy-ERH group); the other line was located along the middle hepatic vein (Cantlie's line) and separates right and left liver lobe (right hemihepatectomy-RH group).

Hypertrophy interval (HI) referred to the period between the first stage of ALPPS and the second volumetry (days). Interstage interval (ISI) referred to the period between the first and second stages of ALPPS (days). Total liver volume (TLV) represented the volume of the whole liver, including the tumor volume. Future liver remnant (FLR) presented the part of the liver to be preserved after ALPPS procedure and it was calculated using CT volumetry prior to stage one.

Deportalized liver (DPL) was the part of the liver without portal flow after the first ALPPS stage. The degree of hypertrophy (DH) was defined as the volume increase between the first stage and the second volumetry, expressed in milliliters and percentages. Standardized residual liver volume (sFLR) represented the FLR to TLV volume ratio, expressed in absolute numbers and percentages. For simplicity, FLR/TLV abbreviation is used in the text instead of sFLR. Kinetic growth rate (KGR) was calculated by dividing DH by the period (days) between the first stage and the second volumetry, expressed as a percentage of volume growth per day or in milliliters (total increase in FLR in ml/interval of hypertrophy). Treatment outcomes included postoperative (3-month) mortality, complication rate, 1-year survival, and 1-year recurrence-free survival.

### Volumetric analysis

Radiological images were analyzed using the licensed radiologic software “syngo.via” (manufactured by Siemens Healthineers, Forchheim, Germany). Alternatively, after receiving complete recordings in the DICOM file (from external institutions), the recordings were processed by the first author using the “open-source” software tool “ImageJ2” (National Institutes of Health and the Laboratory for Optical and Computational Instrumentation (LOCI, University of Wisconsin). At least two post-contrast multiphasic CT or MRI scans (preoperatively and before stage two) were performed according to the staging protocol for liver tumors. Volumetric analysis of the venous phase was done by semi-automated volumetry.

Volumes of intrahepatic blood vessels in the part of the liver marked for resection or FLR were included in volumetric calculations, while surrounding extrahepatic blood vessels, portal vein, inferior vena cava, gallbladder, and intrahepatic tumor were excluded. The process was assisted by the radiologist specialized in abdominal and liver imaging. Liver outline was manually segmented on contrast CT in the axial planes (1 mm slice thickness), with delineation on every third slice. Volumetric results were calculated using software tools following manual delineation of liver boundaries. The same manual delineation procedure was performed for FLR and DPL. Finally, the transection plane was defined in consensus by an experienced liver surgeon and radiologist.

### Statistics

The data were prepared using Microsoft Office Excel (version 16.0, 2016). Statistical analyses were conducted using commercially available IBM SPSS Statistics, version 25.0 (Chicago, IL SPSS In), and results were graphically presented using licensed GraphPad Prism 8 (Dotmatics. © 2023). The Kolmogorov-Smirnov test was utilized to assess the normality of data distribution.

The Student’s *t*-test was applied for continuous data with a normal distribution; otherwise, the Mann-Whitney U test was used. The chi-square test was employed for comparisons between categorical variables. The Wilcoxon Cox test was used to determine the significance of volume increase between two CT volumetries. Pearson's test was used for correlation analysis. A *p*-value <0.05 was considered significant. Logistic regression was employed to identify independent risk factors for severe morbidity/mortality. One-year survival and one-year recurrence-free survival were depicted graphically via Kaplan Meier curve, and results were compared using the log-rank test.

## Results

**Figure 1:**
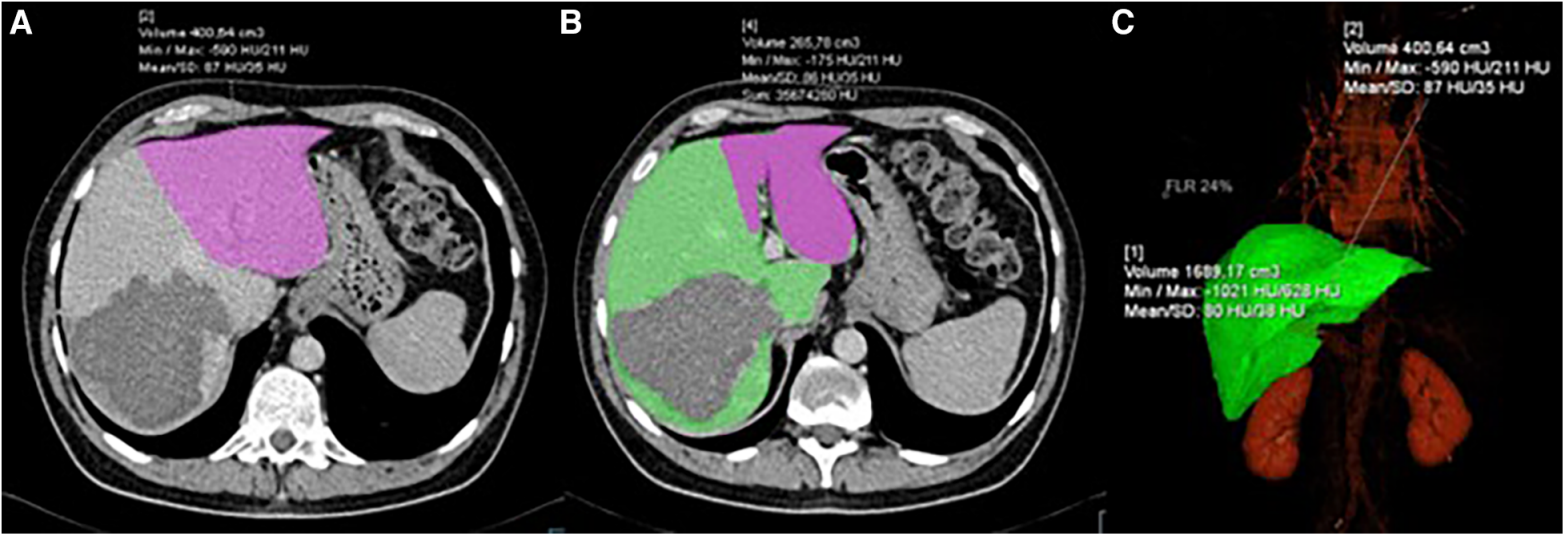
CT volumetry process showing: (**A**) delineation and volume calculation of FLR (pink); (**B**) delineation of FLR (pink) and DPL (green); (**C**) 3D reconstruction of the liver with volume calculation.

A cohort of 143 patients was analyzed from January 1, 2015, to December 31, 2023. Seven patients (4.8%), four in the ERH group and three in the RH group, failed to reach stage 2 due to death during the interstage period. Five of these patients died as a result of septic complications, and two due to liver failure. Six of them did not undergo the second volumetry. Therefore, our analysis included 136 patients, with 72 in the ERH group and 64 in the RH group. Demographic and clinical data are presented in Table 1. Men accounted for 64.7% (88/136), with a median age of 58.2 (±11.5) years, ranging from 24 to 76. Age, gender, body weight, and height did not significantly differ between the groups. Neoadjuvant chemotherapy for CRC was administered in 75% of cases. The rectum was the primary site in 21.3%. The primary tumor site and the rate of liver parenchymal diseases did not statistically differ between the groups. Volumetric characteristics are presented in Table 1. Preoperative volumetry using CT scan was conducted in 124 (91.1%) patients, and MRI was a diagnostic modality in 12 patients. The second volumetry was performed within 6–13 days, with no significant differences in HI length [*t*(134) = −1.22, *p* = 0.22] or ISI (*p* = 0.46). Main part of volumetric process involving FLR and DPL delineation and shadowing is shown in Figure 1.

**Table 1:** Clinical and operative characteristics.

Group	ERH	RH	*p*-value
Antropometric data			
Number of cases	72	64	** **
Age	58.2 ± 11.7	59.9 ± 11.4	0.61
Sex (M/F)	46/26	43/21	0.68
Body weight (kg)	73.1 ± 12.7	75.9 ± 14.8	0.23
Body surface area (m^2^)	1.82 ± 0.23	1.85 ± 0,21	0.22
Body mass index	24.7 ± 3.7	25.1 ± 4.2	0.24
Tumor characteristics			
Primary tumor (rectum/colon)	16/56	13/51	0.83
Chemotherapy (yes/no)	53/19	49/15	0.84
Synchronous metastases (yes/no)	40/32	33/31	0.73
Number of lesions (median, range)	8 (4–13)	9 (3–12)	0.89
CRLM in FLR (%)	12/72 (16.6%)	46/64 (71.8%)	**<0**.**01**
Diameter of largest lesion (median, IQR)	60 (40–99)	68 (34–110)	0.48
Fong score (mean ± SD)	2.8 ± 0.8	3.1 ± 0.9	0.34
Characteristics of volumetry			
Preoperative volumetry (CT/MRI)	66/6	58/6	0.98
Intervolumetric interval (days)	29 ± 9.0	27 ± 8.7	0.19
Hypertrophy interval (days)	7.64 *± *1.2	7.40 *± *1.1	0.22
Interstage interval (days)	10.8 ± 3.1	11.2 ± 3.2	0.46
Second volumetry (CT/MRI)	70/2	62/2	0.99
Operative characteristics			
Surgery duration—first stage (min)	311 (±109)	330 (±119)	0.33
Surgery duration—second stage (min)	145 (±72)	155 (±88)	0.46
Pringle manoeuvre used (%)	46/72 (63.8%)	45/64 (70.0%)	0.34
Blood loss (>500 ml)—first stage	25/72 (34.7%)	21/64 (32.8%)	0.46
Blood loss (>500 ml)—second stage	8/72 (11.1%)	10/64 (15.6%)	0.45
Clean-up in FLR	17/72 (23.6%)	26/64 (40.6%)	**0**.**04**
R0 resection	65/72 (90.2%)	59/64 (92.1%)	0.69
Liver disease (steatosis or fibrosis)	8/72 (11.1%)	10/64 (15.6%)	0.43

Bold indicates values that are statistically significant.

M, male; F, female; FLR, future liver remnant; CT, computed tomography; CRLM, colorectal liver metastasis; MRI, magnetic resonance imaging.

**Figure 2:**
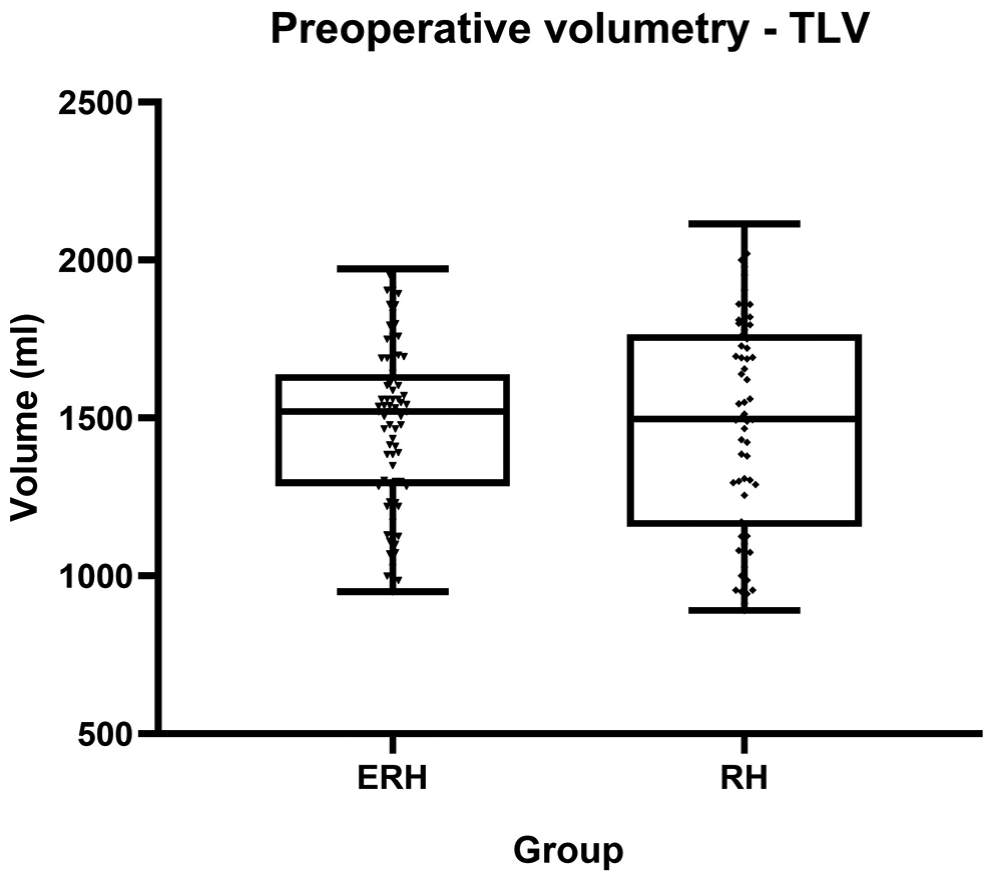
Box plot diagram showing preoperative TLV.

**Figure 3:**
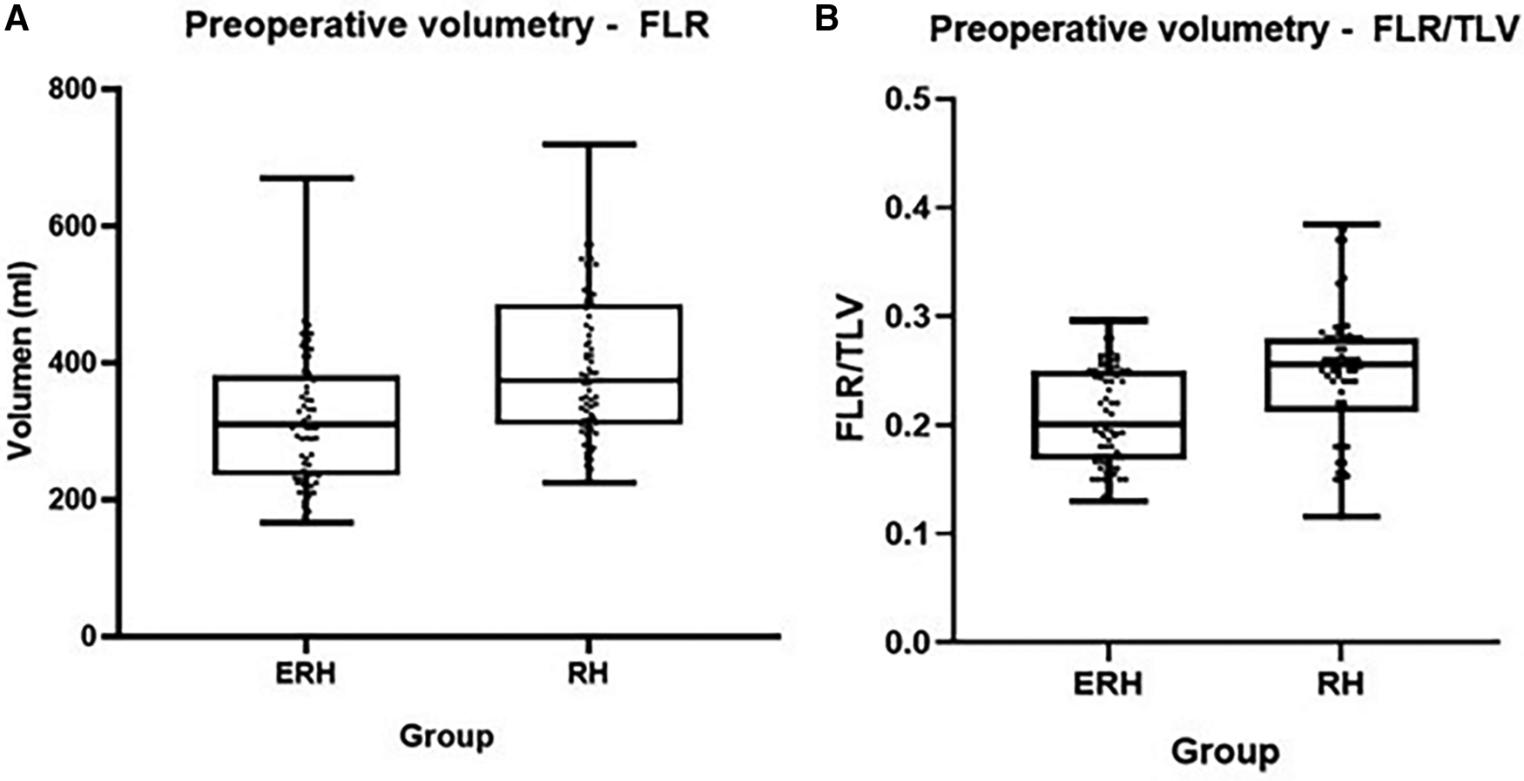
Box plot diagrams showing preoperative FLR (**A**) and preoperative FLR/TLV (**B**).

The average duration of the first and second stages was 318 min (±114) and 151 min (±86), respectively. Although the length of the first stage was longer in the RH group, it did not reach statistical significance (*p* = 0.33). “Clean-up” in FLR during the first stage was more common in the DH group (40.6% vs. 23.6%, *p* = 0.04). Other operative characteristics, including blood loss, the use of the Pringle maneuver, and the duration of the second stage, did not show statistically significant differences. The median TLV for the ERH and RH groups was 1,520 ml (1,285–1,639) and 1,493 ml (1,155–1,765), respectively. Comparative analysis (Table 2; Figures 2, 3) did not show a significant difference in TLV (*p* = 0.9), in contrast to FLR (*p* < 0.01) and DPL volume (*p* < 0.01).

**Table 2:** Preoperative volumetry results.

	TLV (ml), median (IQR)	FLR (ml), median (IQR)	FLR/TLV	FLR/BW, median (IQR)	DPL (ml), median (IQR)
ERH	1,520 (1,285–1,639)	310 (240–388)	0.20 (0.16–0.25)	0.42 (0.34–0.49)	1,130 (971–1,318)
RH	1,493 (1,155–1,765)	383 (307–488)	0.25 (0.20–0.28)	0.49 (0.41–0.55)	985 (861–1,221)
*p*-value	0.90	**<0.01**	**<0.01**	**<0.01**	**<0.01**

Bold indicates values that are statistically significant.

TLV, total liver volume; FLR, future liver remnant; DPL, deportalized lobe; BW, body weight; IQR, interquartile range.

**Figure 4:**
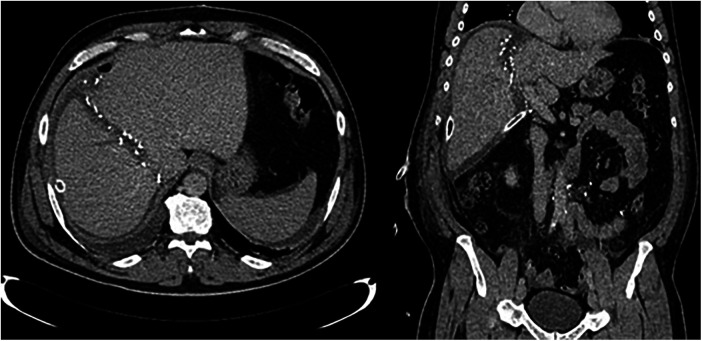
CT images after stage one showing partitioned liver and surgical clips along the transection line.

**Figure 5:**
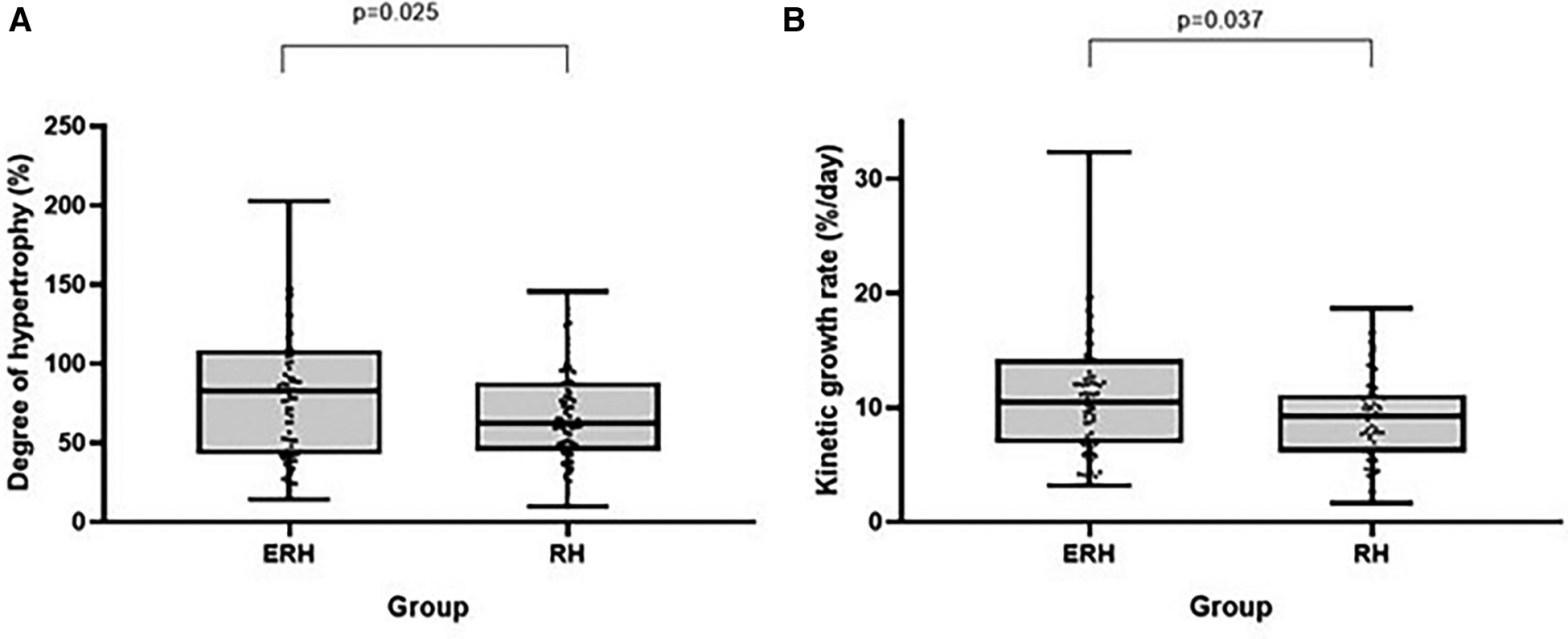
Box plots showing comparison of DH (**A**) and KGR (**B**) between the groups.

The results of the second volumetry are presented in Table 3, with a second CT example of the liver transection line shown in Figure 4. Both groups experienced a significant increase in FLR volume (absolute and relative) between the two stages (Table 4; Figure 5). Additionally, both groups showed a significant increase in TLV and a significant decrease in DPL volume.

**Table 3:** Second volumetry following hypertrophy interval.

	TLV (ml)	FLR (ml)	FLR/TLV	FLR/BW	DPL (ml)
ERH	1,620.3 (1,502–1,877)	556.0 (432–706)	0.37 (0.30–0.44)	0.75 (0.65–0.83)	985 (852–1,145)
RH	1,680.0 (1,481–1,969)	658.0 (512–760)	0.43 (0.34–0.49)	0.80 (0.72–0.87)	890 (796–985)

TLV, total liver volume; FLR, future liver remnant; DPL, deportalized lobe; BW, body weight; IQR, interquartile range; Values are shown as median and IQR.

**Table 4:** Changes of volumetric characteristics during hypertrophy interval.

	FLR increase (ml)	Degree of hypertrophy (%), median (IQR)	Kinetic growth rate ml/day	Kinetic growth rate %/dan	FLR/BW increase (%)
ERH	222.0 (147.0–331.0)	83.2 (41.7–108.2)	29.3 (20.1–41.5)	10.4 (6.8–14.2)	78.5 (45.8–103.2)
RH	249.0 (135.0–348.0)	62.5 (31.7–96.44)	34.1 (17.1–48.9)	9.2 (6.2–11.7)	63.2 (36.5–93.4)
*p*-value	0.46	0.025	0.38	0.037	0.041

FLR, future liver remnant; BW, body weight; Values are shown as median and IQR.

ALPPS in the ERH group resulted in a significantly higher degree of hypertrophy (83.2% vs. 62.5%; *p* = 0.025) and kinetic growth rate (10.4 vs. 9.2; *p* = 0.037). To a lesser extent, the difference was still considerable when comparing the FLR/BW increase between the two stages (78.5% vs. 63.2%; *p* = 0.041).

**Figure 6:**
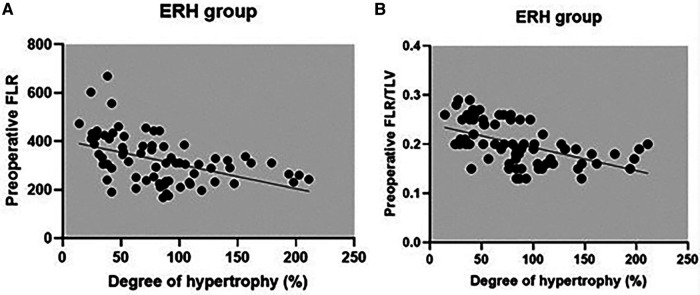
Correlation between preoperative FLR (**A**), preoperative FLR/TLV (**B**) and DH for ERH group.

**Figure 7:**
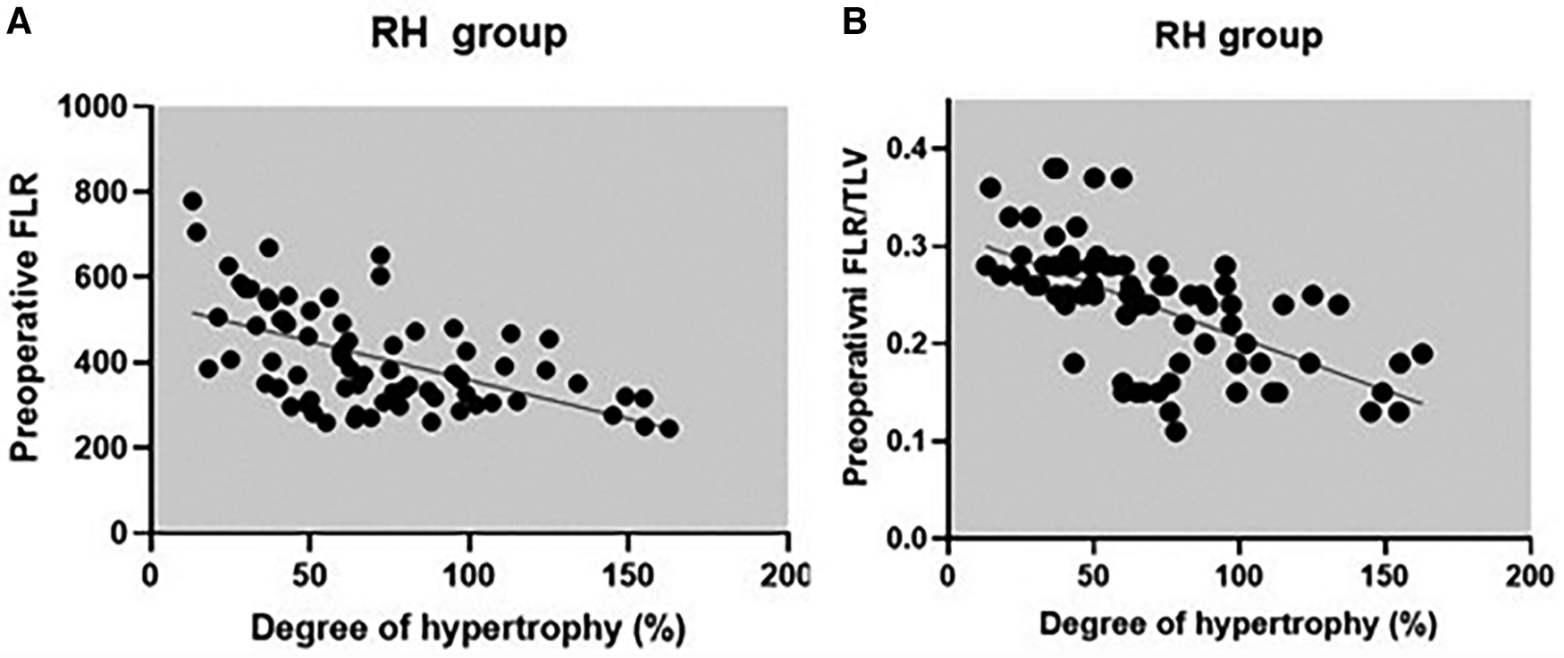
Correlation between preoperative FLR (**A**), preoperative FLR/TLV (**B**) and DH for RH group.

The correlation between preoperative volumetric variables (FLR and FLR/TLV) and the degree of FLR hypertrophy is shown in Table 5 and scatter plots (Figures 6, 7). Both groups demonstrated a strong negative correlation between the initial FLR and DH (*r* = −0.5580, dF = 166, *p* < 0.0001) and between FLR/TLV and DH (*r* = −0.6022, dF = 166, *p* < 0.0001).

**Table 5:** Correlation of preoperative volumetric parameters and degree of hypertrophy.

	Preoperative FLR vs. DH	Preoperative FLR/TLV vs. DH
Overall	(*r* = −0.52, dF = 134, *p* = <0.0001)	(*r* = −0.58, dF = 134, *p* = <0.0001)
ERH	(*r* = −0.46, dF = 70, *p* = <0.0001)	(*r* = −0.51, dF = 70, *p* = <0.0001)
RH	(*r* = −0.54, dF = 62, *p* = <0.0001)	(*r* = −0.59, dF = 62, *p* = <0.0001)

FLR, future liver remnant; TLV, total liver volume; *r*, pearson coefficient.

**Figure 8:**
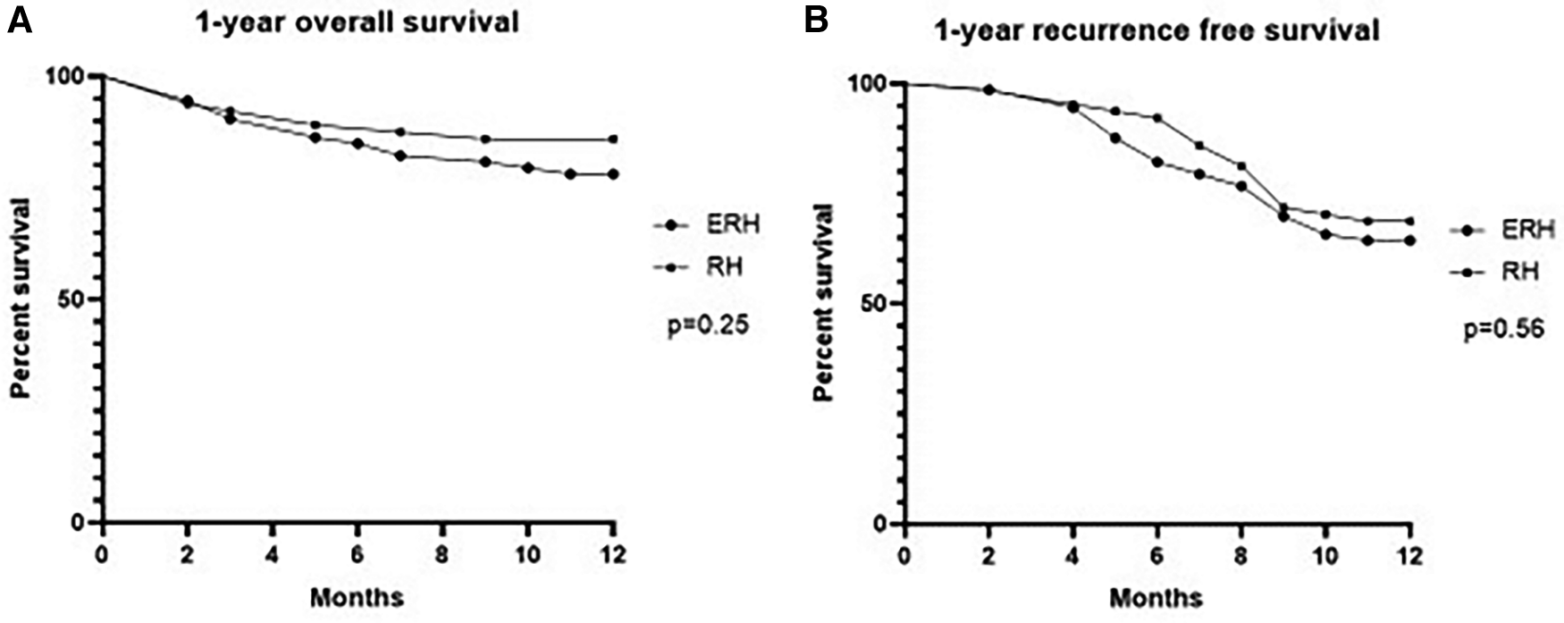
Kaplan–Meier curves showing one-year survival (**A**) and one-year recurrence survival (**B**).

Treatment outcomes are demonstrated in Table 6. The overall mortality rate was 8.8%, and it was comparable between the groups. Similarly, there was no difference in the complication rate and one-year outcomes. Bile leak was observed in nine cases, with six occurring in the RH group. One-year overall and recurrence-free one-year survival are presented in Table 6 and graphically as Kaplan-Meier curves in Figure 8.

**Table 6:** Postoperative outcomes.

	Mortality (3 months)	Complication rate (>3a*)	PHLF**	Bile leakage	1-year survival	1-year recurrence-free survival
Overall	12/136 (8.8%)	33/136 (24.2%)	20/136 (14.7%)	9/136 (6.6%)	112/136 (82.3%)	91/136 (66.9%)
ERH	7/72 (9.7%)	17/72 (23.6%)	12/72 (16.6%)	3/72 (4.1%)	57/72 (79.1%)	47/72 (65.2%)
RH	5/64 (7.8%)	16/64 (25.0%)	8/64 (12.5%)	6/64 (8.2%)	55/64 (85.9%)	44/64 (68.7%)
*p*-value	0.69	0.85	0.49	0.22	0.25	0.56

PHLF, post-hepatectomy liver failure.

* According to Clavien–Dindo classification.

** According to 50-50 criteria.

## Discussion

In 2020, in the European Union, CRC was the second most frequent malignant disease, accounting for 12.7% of all malignancies and 12.4% of all deaths due to cancer (10). Five-year survival for CRC diagnosed between 2010 and 2014 in Croatia was 51.5% (11). Survival is over 90% for localized disease (T1–T2, N0), but fewer than 35% of colorectal cancers are found at this stage of the disease (12). Unfortunately, at the time of diagnosis, liver metastases are already present in 15%–25% of patients (1).

The treatment of bilobar CRLMs is complex and challenging, particularly for surgeons who should be very familiar with anatomical and functional liver characteristics. Beside anatomical considerations, the capacity of postoperative liver regeneration and the risk factors for PHLF should be assessed as part of preoperative planning. The CRLM resectability mostly depends on the preservation of adequate FLR. Techniques for FLR hypertrophy stimulation increase the resectability of CRLMs and other liver tumors. ALPPS has recently become the standard part of surgeons’ armamentarium in many hepatobiliary centers. Previous research has shown significant variability in FLR hypertrophy following ALPPS, prompting investigations into various technical aspects and disease factors influencing outcomes. Therefore, many authors studied technical ALPPS characteristics, liver parenchymal condition, and patient comorbidities as potential factors of FLR hypertrophy (2, 3, 8, 13–16). However, the impact of the liver transection line on FLR hypertrophy has not been thoroughly explored, and understanding the dynamics of FLR hypertrophy is critical for optimizing surgical outcomes and minimizing complications.

Differences in hypertrophy dynamics of FLR of different sizes were investigated after partial hepatectomies, living liver donors, and partial liver graft recipients. These demonstrated that regeneration depends on body mass, TLV, and FLR (17–19). In addition, the liver regenerates faster in the recipient than in the donor, which applies to all grafts (left lobe, right lobe, and left lateral section). Regeneration is also associated with operative characteristics such as middle hepatic vein preservation (20).

Modern imaging-based volumetry began in the 1990s, mainly due to the evolution of PVE and major liver resections (21). The main goal of volumetry in liver surgery is accurate FLR determination to prevent PHLF. Volumetry can be improved with digital tools for automated volumetrics. Digital tools not only detect liver contours and measure regional and segmental liver volumes, but also facilitate 3D liver visualization, virtual reality, and holographic simulations. In this way, such tools allow for more precise resections and the maximum preservation of healthy liver parenchyma (22).

Initially, manual volumetry was the only technique for volume calculations. It included manual delineation of the liver contours on each slice surface at axial cross-sections. The desired volume was the sum of all the measured surfaces. This method was time-consuming and difficult to master. Therefore, volumetry has become automated or semi-automated following the development of advanced digital tools and the improvement of radiological platforms. In automated volumetrics, more complex digital tools, including artificial intelligence, are used for volume calculations. The main advantage is speed. Disadvantages include high costs and difficulties recognizing liver contours when an organ, tumor, or tissue with similar CT characteristics surrounds the liver. Consequently, semi-automated volumetry is the most commonly used in clinical practice. Semi-automated volumetry requires the assistance of digital tools, but the primary stages of measurement (delineation and tumor contour definition) are under human control. Volumetry can be performed on both MRI and CT scans, and measurement precision is comparable between the two methods. It is however, recommended to use the same imaging technique for both volumetries, as this should maximize the reliability of the results.

A cohort of 136 consecutive patients represented a significant sample, given that ALPPS is a procedure with narrow indications performed in highly specialized centers. In both groups, men were more common (64.7%), which is expected given that primary CRC is 30% more frequent in men. The mean number of CRLMs and Fong score (23), the most common prognostic score for CRLM, did not differ between the groups. These disease characteristics did not impact the transection line itself (as it depends on localization of CRLMs rather than size/number), however, this was important for the secondary outcome: comparison of 1-year oncological outcomes between the two groups.

The length of HI and ISI followed recommendations, mostly from 6 to 9 days for HI and 7–12 days for ISI. Although some authors recommend prolonging the interval between the two stages to 3–4 weeks, most support the thesis that a prolonged waiting period may increase the risk of tumor growth and metastasizing (3, 8, 24, 25).

Second volumetry showed a significant TLV increase in both groups. Still, it did not solely correspond to the increase in FLR volume since there was a DPL atrophy during the HI. This atrophy was not so intense when compared to FLR hypertrophy. However, the results confirmed the significance of the atrophy-hypertrophy complex (AHC) as a basic principle in liver hypertrophy stimulation (26). AHC explains that in cases of re-routing portal flow from one liver part to another, there are always two synchronous processes: atrophy of deportalized and hypertrophy of the portalized liver part. A similar process happens when there is hepatic artery occlusion or biliary outflow occlusion, but this topic is beyond the scope of this paper.

Our results indicate that localization of the liver transection line is associated with different degrees of FLR hypertrophy, but the next question is: what is the cause of such difference? Factors in PVE partly explain the mechanisms of liver hypertrophy in ALPPS. Still, the exact mechanisms of faster hypertrophy, when compared to PVE or liver regeneration after standard resections, are a matter of debate. Anatomically and technically, the portal vein occlusion is the same in both methods. Still, the important difference is liver transection and disruption of the portal collaterals between the two liver parts in ALPPS. Such surgical trauma, along with physiological changes and local/systemic inflammatory processes represent the main differences between the two procedures. Our study confirmed rapid and intensive FLR hypertrophy in ALPPS for both groups, which is similar to the results of previous studies that demonstrated ALPPS efficiency in inducing FLR hypertrophy (4, 7, 27–29). However, we also showed that the DH depends on the localization of the liver transection line. More intensive hypertrophy was seen in the ERH group (median DH of 83% vs. 62% and median KGR of 10.4 ml/day vs. 9.2 ml/day), as this group had smaller preoperative FLR and FLR/TLV.

Several conclusions can be drawn when analyzing volumetric changes during HI, as presented in Table 4. The liver transection line in the ERH group resulted in a smaller liver volume perfused with portal blood when compared to RH group. In addition, the DPL was larger in the ERH group, and FLR/TLV was smaller in the ERH group. Finally, correlation analyses showed that smaller FLRs hypertrophied more intensively (faster and to a greater extent). An even stronger negative correlation was found between FLR/TLV and DH. In summary, the smaller the preoperative FLR (and FLR/TLV), the higher the degree of FLR hypertrophy. This also suggests that FLR hypertrophy is favored by the greater volume of the DPL, a phenomenon already observed in PVE (30).

We found only one study on the correlation between preoperative FLR and DH that investigated hemodynamic liver changes during ALPPS (31). FLR hypertrophy negatively correlated with the initial FLR/TLV and FLR/BM. The main drawback was the small sample size (23 patients) with no stratification according to the liver transection line. Thus, the volumetric results from our study may be the starting point for future analysis of DH variations in ALPPS related to liver transection lines.

In many studies, an animal model was established to investigate the mechanisms of FLR hypertrophy in ALPPS (32–34). A prevailing common conclusion of these studies is that both humoral and hemodynamic mechanisms contribute to FLR hypertrophy. Humoral mechanisms result from surgical trauma, liver transection, and systematic inflammatory response, releasing proinflammatory factors such as IL-6 and TNF-α (32, 35). It remains questionable if “clean-ups” in the RH group could produce a more pronounced humoral response, keeping in mind that every surgical trauma or resection results in an inflammatory response. However, we consider that hemodynamic changes, particularly the redirection of portal blood flow into the FLR, play a pivotal role in promoting FLR hypertrophy. Therefore, differences in FLR hypertrophy between the ERH and RH groups may stem from variations in portal hemodynamics following liver transection. As a result of stage one, the whole portal blood is redirected into FLR. This increases portal pressure and portal flow per gram of liver, which is mainly determined by the FLR and DPL volumes. This thesis is supported by previous studies (4, 34, 36). It is worth mentioning that the impact of partial ALPPS on FLR hypertrophy was also studied, and results are somewhat contradictory. While most authors suggest that partial ALPPS is associated with similar FLR hypertrophy and lower morbidity when compared to complete ALPPS (2, 37), others did not confirm the superiority of partial ALPPS in animal models (with regards to FLR hypertrophy capacity) (38). On the other side, liver steatosis or cirrhosis seem to delay the liver regeneration process during ALPPS (16, 39).

During the early phases of ALPPS development, the studies mainly focused on the volume and explanation of surprisingly rapid and intense FLR hypertrophy. Technical improvements, optimal patient selection, and referral of patients to high-volume centers significantly improved early postoperative outcomes (40). However, PHLF frequently develops after seemingly adequate FLR hypertrophy. Consequently, the function of the hypertrophied FLR was questioned (27). Studies using hepatobiliary scintigraphy (41), LIMAx test (42) and indocyanine green retention test (43) resulted in the same conclusion: the increase in FLR volume is not followed by a proportional increase in liver function. Thus, it is advocated by some authors to consider using these tests to evaluate FLR function before proceeding with the second stage of ALPPS.

Despite the different degrees of liver resection, no significant differences were detected when evaluating early and 1-year postoperative outcomes. It can be explained by the main results of our study which indicate that initially a smaller FLR is compensated by more intensive DH in ERH group. Although, clinical postoperative and oncologic outcomes were not primary endpoints of this study, the results on morbidity/mortality and 1-year survival rates provide some important insights. Principally, we showed that localization of transection line (or more extensive liver resection) did not have impact on postoperative and 1-year outcomes.

Overall, the results presented contribute to the understanding of postoperative liver hypertrophy in ALPPS, which might change surgical planning and improve the prediction of FLR hypertrophy even before the first stage, which is crucial for optimal patient selection. The treatment approach should be individualized since when estimating FLR hypertrophy in each individual patient, many factors should be considered (such as liver condition, tumor type, liver volume, and comorbidities), including a planned liver transection line. Finally, PVE was standard of care to increase the FLR for a long period, and in the last decade, ALPPS challenged this paradigm. Yet, the role of ALPPS should be reconsidered in light of recent development of liver venous deprivation (LVD) (44). The LVD technique is based on concomitant portal vein and hepatic vein embolization in the diseased part of the liver. The technique is minimally invasive, and the resulting hypertrophy of the FLR is superior to PVE and comparable to ALPPS (44, 45). However, LVD requires scientific and clinical evaluation on larger cohorts so optimal indications are still a matter of debate. Future comparative studies will show us if LVD can completely replace ALPPS and PVE.

Our study has several limitations. First, it is non-randomized since patients were treated according to defined surgical indications depending on the distribution of CRLMs. Second, we could not examine molecular and hemodynamic mechanisms as causes of FLR hypertrophy, and all conclusions were based on volumetric liver changes and, to a lesser extent, on clinical and intraoperative parameters. Third, given that the main aim was to analyze the liver transection line and FLR hypertrophy, we did not analyze the possible impact of postoperative complications on FLR hypertrophy in the first stage. In the end, not all surgical procedures in this cohort were done by the same surgeon, but in inclusion criteria, we limited participants only to complete, open ALPPS as described by the first author (Hans Schlitt). Various ALPPS variations were excluded as these may have had an effect on FLR hypertrophy. Minor differences in technique such as type of liver resection plane covering (e.g., sterile bag or Tachosil) or technique of liver transection (e.g., CUSA; clamp-crush technique), were inevitable, but these factors were not expected to affect FLR hypertrophy.

## Conclusions

ALPPS provides valuable insights into postoperative liver hypertrophy dynamics. Our findings highlight the impact of the liver transection line on future liver remnant (FLR) hypertrophy. Right trisectionectomy was associated with more intensive FLR growth compared to right hemihepatectomy. Additionally, we observed a negative correlation between FLR volume and the degree of hypertrophy (DH). ALPPS remains a crucial strategy for managing bilobar colorectal cancer liver metastases (CRLMs) with inadequate FLR volume. Larger studies are warranted to elucidate the mechanisms driving FLR hypertrophy and refine surgical strategies in CRLM management. The reliability of CT volumetry for liver volume analysis underscores its indispensable role in liver regeneration research and preoperative assessment of CRLM resectability.

## Data Availability

The original contributions presented in the study are included in the article/Supplementary Material, further inquiries can be directed to the corresponding author.
